# Capsaicinoid Glucoside Attenuates Lipid Accumulation in HepG2 Cells Through TRPV1/AMPK‐Dependent Signaling Pathway

**DOI:** 10.1002/fsn3.70564

**Published:** 2025-07-02

**Authors:** Abdeen Elkhedir, Alsadig Yahya, Eihab H. Jadelrab, Mazin Mohamed Salih, Amgad Albahi, Yassin Haran, Mamoun A. Homaida, Hammad Hamed, Xiaoyun Xu

**Affiliations:** ^1^ Agro‐Industries Institute, Industrial Research and Consultancy Centre (IRCC) Khartoum Sudan; ^2^ Food Science and Technology College of Agricultural Studies, Sudan University of Science & Technology Khartoum Sudan; ^3^ Department of Food Science and Technology, Faculty of Agriculture and Natural Resources University of Bakht Al‐Ruda Ed Dueim Sudan; ^4^ National Food Research Centre Ministry of Agriculture and Natural Resources Khartoum Sudan; ^5^ College of Food Science and Technology Huazhong Agricultural University Wuhan China

**Keywords:** AMPK, Capsaicinoid glucoside, cellular signaling pathway, HepG2 cells, lipid metabolism, TRPV1

## Abstract

Metabolic disorders associated with excessive lipid accumulation pose a significant global health challenge. Capsaicinoid glucoside (CG), a novel water‐soluble derivative of capsaicin, offers improved bioavailability and reduced pungency. In this study, we evaluated the lipid‐lowering effects of CG and its underlying mechanisms in oleic acid (OA)‐induced HepG2 cells. Treatment with 100 μg/mL CG significantly reduced triglyceride (TG) levels by 60.7% and total cholesterol (TC) levels by 34.4%, compared to the OA group. CG also markedly decreased intracellular reactive oxygen species (ROS) and reduced lipid droplet accumulation, as confirmed by Oil Red O staining. At the molecular level, CG significantly upregulated TRPV1 and AMPK gene and protein expression, while downregulating lipogenic markers such as SREBP‐1c and FASN. It also increased the expression of lipid oxidation‐related genes, including PPARδ. Molecular docking revealed stable interactions of CG with both TRPV1 and AMPK, supporting their roles in the observed metabolic effects. These findings suggest that CG exerts its lipid‐lowering and antioxidant effects via the TRPV1/AMPK signaling pathway and highlight its potential application as a functional food component or therapeutic supplement for improving hepatic lipid metabolism.

## Graphical abstract

Capsaicinoid glucoside (CG), reduces triglyceride (TG) and total cholesterol (TC) accumulation in OA‐treated HepG2 cells. CG alleviates reactive oxygen species (ROS) and enhancing lipid oxidation. CG activates the TRPV1/AMPK pathway, while downregulating the expression of lipogenic genes SREBP‐1C and FAS. Molecular docking indicates that CG forms stable complexes with TRPV1 and AMPK, suggesting their involvement in lipid regulation.

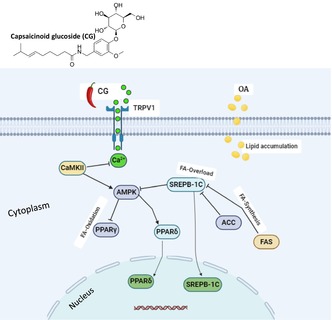

## 1 Introduction

The global rise in modern lifestyle‐associated chronic diseases such as obesity has increased the need for effective therapeutic strategies (Balwan and Kour 2021; Bodai et al. 2018; Singh et al. 2022). Obesity is one of the fastest‐growing health problems in the world (Ng et al. 2014), with many common diseases such as diabetes and cardiovascular disease closely linked to being overweight and obese (Gjermeni et al. 2021). Additionally, obesity, characterized by excessive buildup of body fat, has spread worldwide like an epidemic (Gerges et al. 2021). Numerous metabolic comorbidities, including fatty liver, cardiovascular disease, type‐2 diabetes, neurological disorders, and even some malignancies, are strongly associated with excessive fat deposition due to dysfunctional lipid metabolism and chronic inflammation (Gesta et al. 2007; Hotamisligil 2006).

While various natural compounds—including polysaccharides (e.g., fucoidan), peptides (e.g., soy‐derived), and polyphenols (e.g., resveratrol)—have shown promise in modulating lipid metabolism (Poulios et al. 2024), capsaicinoids from chili peppers (*Capsicum* spp.) represent particularly compelling candidates due to their multimodal mechanisms of action (Joshi et al. 2024; Poulios et al. 2024). Capsaicinoids, the most important class of these phytochemicals, are naturally occurring pungency ingredients in peppers (*Capsicum* spp). According to current knowledge, capsaicinoids have a wide range of biological and physiological actions, such as reducing neuropathic pain and inflammation (Choi et al. 2011), reducing oxidative stress (Elkhedir et al. 2022; Srinivasan 2016), and providing benefits for conditions including anti‐lithogenic effect, diabetic neuropathy, psoriasis, cardioprotective effects, arthritis, and cancer (Yang et al. 2010). Capsaicinoids may also help in weight loss by activating receptors, promoting lipolysis, stimulating thermogenesis, and increasing energy expenditure (Whiting et al. 2012). Additionally, studies have shown that capsaicinoids can prevent non‐alcoholic fatty liver disease (NAFLD) by promoting lipolysis and reducing lipid deposition in mouse liver (Hu et al. 2017). Furthermore, capsaicinoid consumption may stimulate lipid autophagy in hepatocytes, which is directly associated with lipogenesis (Bort et al. 2019; Shin et al. 2020).

Mechanistically, capsaicinoids exert many of their metabolic effects via the transient receptor potential vanilloid 1 (TRPV1) channel, a non‐selective cation channel initially identified as the receptor responsible for capsaicin's pungency (Shuba 2021). In parallel, AMP‐activated protein kinase (AMPK) is a critical regulator of cellular energy homeostasis and lipid metabolism, modulating key pathways involved in lipogenesis and lipid oxidation (Viollet and Andreelli 2011).

Despite their promising bioactivity, the application of capsaicinoids is limited by several drawbacks, including poor water solubility, low bioavailability, and pungency, which hinder their use in food and pharmaceutical formulations. In contrast, capsaicinoid glucoside (CG), a novel glycosylated derivative, offers improved solubility and tolerability while maintaining or even enhancing the biological activity of the parent compound (Elkhedir et al. 2022).

Various approaches have been proposed to mitigate the abnormal buildup of adipose tissue associated with obesity. In this study, we examined whether CG regulates lipid metabolism and explored the underlying mechanism.

## 2 Materials and Methods

### 2.1 Preparation of Capsaicinoid‐Derivatives

The capsaicinoid‐derivatives were isolated and characterized using UPLC‐QTOF‐MS according to our previous study (Elkhedir et al. 2022, 2024). Briefly, capsaicinoid‐derivatives were diluted in dimethyl sulfoxide (DMSO) for treatment of HepG2 cells and stored at −20°C as a stock solution. Then, appropriate concentrations of test dose were freshly prepared each time from the stock solutions in MEM medium.

### 2.2 Cell‐Culture and Treatment

The HepG2‐cell line, originating from human hepatocellular carcinoma, was obtained from the Stem Cell Bank of the Chinese Academy of Sciences in Shanghai, China. These cells were maintained under controlled conditions at 37°C in a humidified atmosphere containing 5% carbon dioxide (CO_2_). The culture medium used was Dulbecco's Modified Eagle Medium (DMEM), enriched with 10% fetal bovine serum (FBS) and a 1% (v/v) penicillin–streptomycin antibiotic mixture. The medium was replaced every 2–3 days to ensure optimal growth. Upon reaching 80%–90% confluency, the cells were subcultured using a trypsin/EDTA solution, followed by the addition of complete Minimum Essential Medium (MEM) to deactivate the trypsin/EDTA and resuspend the cells. For experimental treatments, the cells were incubated in serum‐free EMEM medium.

### 2.3 Effect of Capsaicinoid‐Derivatives on the Viability of HepG2 Cells

Cells were seeded into 96‐well plates at a density of 1 × 10^4^ cells per well and incubated for 24 h to facilitate adherence. After this period, the culture medium was removed, and the cells were exposed to different concentrations of CG (ranging from 0 to 150 μg/mL) prepared in MEM medium. Following a 24‐h incubation, the treatment solutions were aspirated, and 120 μL of MTT (3‐(4,5‐dimethylthiazol‐2‐yl)‐2,5‐diphenyltetrazolium bromide) solution (0.5 mg/mL) was added to each well (Hu et al. 2020). The plates were then incubated for an additional 3 h to promote the formation of MTT formazan crystals. The MTT solution was subsequently discarded, and 150 μL of dimethyl sulfoxide (DMSO) was introduced into each well to dissolve the formazan crystals. The plates were gently agitated for 5 min to ensure thorough solubilization. Absorbance readings were taken at 490 nm using a microplate reader (MultiScan Go, Thermo Scientific Co. Ltd., Waltham, MA, USA) as re. Each treatment condition was replicated six times. Cell viability was calculated using the specified formula and expressed as a percentage relative to the control group.

$$\%\text{Cell viability}=\frac{\mathrm{OD}\;\text{sample}-\mathrm{OD}\;\text{blank}\;}{\mathrm{OD}\;\text{control}-\mathrm{OD}\;\text{blank}}\times 100$$

### 2.4 Establishment of OA‐Induced Lipids in HepG2 Cells

The HepG2 cells were inoculated in 96‐well plates at a density of 2 × 10^4^ cells/mL and incubated at 37°C in a humidified 5% CO_2_ incubator for 24 h. After incubation, the cells were treated with varying concentrations (0, 0.2, 0.4, 0.6 or 0.8 mM) of oleic acid (OA) to determine the optimal concentration (Wang et al. 2025). This was done using MTT, TG, and Oil Red O Stain. To examine the effect of OA‐induced lipids on HepG2 cells viability, the cells were co‐treated with 25, 50, or 100 μg/mL of CG solution in culture media containing 0.4 mM oleic acid for 24 h. Afterward, the cells were exposed to MTT solution (dye dissolved in DMSO) and incubated for 3 h. The cell viability was then calculated using the MTT formula mentioned above.

### 2.5 Measurement of Lipid‐Accumulation by Oil Red O Stain

To assess the accumulation of lipid droplets in HepG2 cells, a freshly prepared Oil Red O Stain solution was used to stain the cells at room temperature for 30 min. After staining, the cells were fixed with a formaldehyde solution for 40 min (Lin et al. 2007). Then, the solution was carefully removed and the cells were rinsed twice with distilled water before being observed under a microscope (Olympus CKX41, Japan).

### 2.6 Intracellular TC and TG Levels

HepG2 cells were plated in 6‐well plates at a density of 2 × 10^5^ cells per well and incubated in a humidified atmosphere containing 5% CO_2_ at 37°C. After 24 h of culture, the cells were treated as described in the previously outlined protocol. Intracellular total cholesterol (TC) and triglyceride (TG) levels were quantified in cell lysates using commercially available TC and TG assay kits. Additionally, the protein concentration in the cell lysates was determined using a bicinchoninic acid (BCA) assay kit, following the manufacturer's guidelines. The TC and TG levels were normalized to cellular protein content and expressed as micromoles of lipid per milligram of protein.

### 2.7 Intracellular ROS Assay

The levels of intracellular reactive oxygen species (ROS) were assessed using 2′,7′‐dichlorodihydrofluorescein diacetate (DCFH‐DA). HepG2 cells were plated in 24‐well plates at a density of 1 × 10^5^ cells per well and cultured at 37°C in a humidified environment with 5% CO_2_. The cells were pre‐treated as outlined in section 2.6. After treatment, they were incubated with DCFH‐DA at a final concentration of 10 μM in culture medium for 30 min (Kong et al. 2023). Post‐incubation, the cells were rinsed three times with phosphate‐buffered saline (PBS) to remove any excess dye. Fluorescence intensity was measured using a 96‐well plate fluorometer (Molecular Devices, SpectraMax M5, CA, USA) with excitation and emission wavelengths set at 485 nm and 535 nm, respectively. For visualization, fluorescence microscopy (ECLIPS Ti; Nikon, Melville, NY, USA) was employed to capture images of the green‐fluorescent cells.

### 2.8 Western Blot Analysis

After the experimental treatment, HepG2 cells were rinsed twice with ice‐cold phosphate‐buffered saline (PBS). The cells were then lysed and homogenized in radioimmunoprecipitation assay (RIPA) buffer supplemented with protease and phosphatase inhibitors. Protein concentrations were determined using a bicinchoninic acid (BCA) protein assay kit. Protein samples were prepared by mixing them with 5X loading buffer in a 4:1 ratio and denatured by heating at 95°C–100°C for 10 min. Once cooled to room temperature, 40 μg of protein from each sample was loaded onto 10% SDS‐polyacrylamide gels (TGX precast gels) and separated by electrophoresis at 80 V for 1.5 h. The resolved proteins were transferred onto polyvinylidene fluoride (PVDF) membranes (0.22 μm, Millipore, USA) using a semi‐dry transfer system. To reduce nonspecific binding, the membranes were blocked with 5% skim milk in Tris‐buffered saline containing 0.1% Tween 20 (TBST) for 2 h at room temperature. After blocking, the membranes were washed three times with TBST (10 min per wash) and incubated overnight at 4°C with the appropriate primary antibodies. Following primary antibody incubation, the membranes were washed five times with TBST (5 min per wash) and incubated with horseradish peroxidase (HRP)‐conjugated secondary antibodies for 2 h at room temperature with gentle shaking. Protein bands were detected using an enhanced chemiluminescence (ECL) detection system and analyzed using Image Studio Software Version 5.2 (LI‐COR, USA).

### 2.9 Molecular Modeling Docking

The interactions between AMP‐activated protein kinase subunit gamma‐1 (AMPK), (https://alphafold.com/entry/Q9Y478), Transient receptor potential cation channel subfamily V member 1 (TRPV1), (https://www.rcsb.org/structure/5IS0), and CG as a ligand were studied using Discovery Studio software (version 2.5) with the LibDock algorithm developed by Accelrys Software Inc. (San Diego, CA, USA). The three‐dimensional (3D) structures of the target proteins were obtained from the RCSB Protein Data Bank. The structures were prepared by removing water molecules and adding hydrogen atoms. Potential binding sites were identified using site‐detection tools to pinpoint concave regions within the protein structures. Energy minimization was conducted using the BEST protocol, which combines best‐Newton minimization in Cartesian space with conjugate‐gradient minimization in torsion space. Molecular docking was performed using the LibDock method, treating the proteins as rigid structures while allowing full flexibility for the small ligand molecule.

### 2.10 Statistical Analyses

Statistical evaluations were performed using an unpaired Student's *t*‐test for analyzing differences between two groups, whereas one‐way analysis of variance (ANOVA) was utilized for comparisons across three or more groups. Data are expressed as mean ± standard deviation (SD). Significant differences between groups were assessed using Duncan's multiple range test, performed with GraphPad Prism 7 (GraphPad Software Inc., San Diego, CA, USA). A *p*‐value of less than 0.05 was considered statistically significant.

## 3 Results

### 3.1 Effect of CG on HepG2 Cells Viability

The MTT assay was employed to assess the appropriate working concentrations of capsaicinoid‐derivatives on HepG2 cells. CG exhibited no significant reduction in cell viability at concentrations ranging from 25 to 100 μg/mL, compared to the control group (*p* < 0.05), as illustrated in Figure S1. Based on these findings, concentrations of 25, 50, and 100 μg/mL of CG extract were selected for pretreatment of the cells.

### 3.2 Effect of CG Derivatives on OA‐Induced Lipids in HepG2 Cells

**FIGURE 1:**
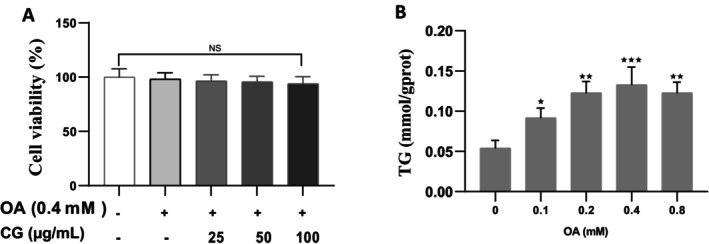
Establishment of OA‐induced lipids in HepG2 cells. (A) Effect of CG on OA‐induced on viability of HepG2 cells. (B) Lipid‐accumulation in OA‐induced HepG2 cells. All experimental data are expressed as means ± SD of at least three independent experiments. *p < 0.05 vs. OA group, *p < 0.05, **p < 0.01, and ***p < 0.001.

To establish the optimal concentration of oleic acid (OA) for inducing lipid accumulation without cytotoxicity, HepG2 cells were treated with 0.1, 0.2, 0.3, 0.4, and 0.8 mM OA. Cell viability remained above 90% at all concentrations, with no significant differences compared to the control (*p* < 0.05). Triglyceride (TG) levels increased in a dose‐dependent manner and peaked at 0.4 mM OA, which was selected for the lipid accumulation model (Figure 1A). Co‐treatment of HepG2 cells with 0.4 mM OA and CG (25, 50, or 100 μg/mL) showed no significant decrease in cell viability (*p* < 0.05), confirming the non‐toxic nature of the treatment combinations (Figure 1B).

### 3.3 Effects of CG on Lipid‐Accumulation in OA‐Treated HepG2 Cells

**FIGURE 2:**
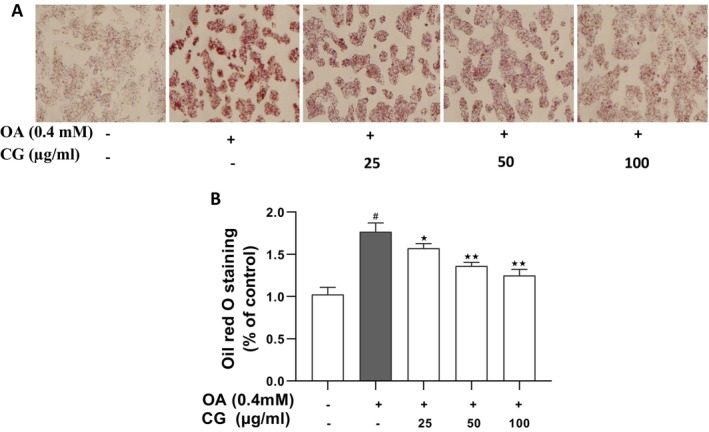
Oil red O staining in OA‐induced HepG2 cells. HepG2 cells were incubated with CG at different concentrations in the presence of 0.4 mM OA for 24 h. (A) Visualization of intracellular lipid droplets in HepG2 cells under microscope. (B) Oil red O staining in the cultured HepG2 cells. All experimental data are expressed as means ± SD of at least three independent experiments. *p < 0.05 vs. OA group, ^#^ *p* < 0.05 vs. HepG2 cells control. *p < 0.05 and **p < 0.01.

Oil Red O staining demonstrated a significant increase in intracellular lipid droplets following OA treatment (*p* < 0.05). In contrast, CG treatment led to a dose‐dependent reduction in lipid droplet number, size, and staining intensity (Figure 2A). Quantification of stained lipids confirmed a significant reduction in CG‐treated groups compared to the OA model group (*p* < 0.05) (Figure 2B).

### 3.4 Effects of CG on TG and TC Contents in AO‐Treated HepG2 Cells

**FIGURE 3:**
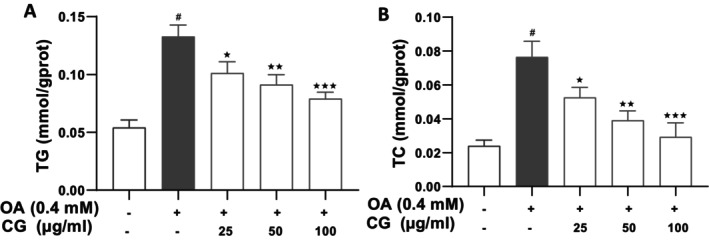
Effect of CG on TG and TC contents of OA‐induced HepG2 cells. HepG2 cells were incubated with different concentrations of CG in the presence of 0.4 mM OA for 24 h. (A) Quantification of intracellular TG contents in HepG2 cells. (B) Quantification of intracellular TC contents in HepG2 cells. All experimental data are expressed as means ± SD of at least three independent experiments. *p < 0.05 vs. OA group, ^#^ *p* < 0.05 vs. HepG2 cells control. *p < 0.05, **p < 0.01, and ***p < 0.001.

CG treatment markedly lowered both TG and total cholesterol (TC) levels in OA‐treated HepG2 cells. At 100 μg/mL, CG reduced TG from 0.13 ± 0.01 mmol/gprot (OA group) to 0.079 ± 0.006 mmol/gprot (*p* < 0.05), representing a 60.7% decrease. Similarly, TC levels decreased from 0.09 ± 0.01 mmol/gprot (OA group) to 0.031 ± 0.004 mmol/gprot (*p* < 0.05), a 34.4% reduction (Figure 3).

### 3.5 CG Inhibited OA‐Induced Intracellular ROS in HepG2 Cells

In healthy cells, a dynamic equilibrium is maintained between the generation and clearance of reactive oxygen species (ROS). At appropriate concentrations, ROS are essential for supporting macrophages in executing their immune‐related functions, such as enzymolysis and phagocytosis (Jia et al. 2018). However, elevated ROS levels can induce cellular damage (Volpe et al. 2018). To measure ROS levels in OA‐induced lipid accumulation in HepG2 cells, dichlorofluorescein (DCF), the oxidized product of H2DCF, was utilized as a detection marker. Compared with the OA‐induced lipid group, the level of ROS significantly increased (*p* < 0.05). However, the level of ROS in the capsaicinoids treated cells group significantly (*p* < 0.05) decreased in a dose‐dependent manner (Figure S2).

### 3.6 Effects of CG on mRNA Expression

Quantitative PCR analysis revealed that OA downregulated TRPV1 and AMPK mRNA expression (*p* < 0.05), while CG (100 μg/mL) restored their levels by 1.5 ± 0.4‐fold and 1.6 ± 0.3‐fold, respectively (*p* < 0.05). Conversely, OA upregulated lipogenic genes (SREBP‐1C, FAS, PPARγ; *p* < 0.05), which were significantly suppressed by CG treatment (*p* < 0.05) (Figure S3).

### 3.7 CG Enhances Expression of Lipid Metabolism‐Related Proteins

**FIGURE 4:**
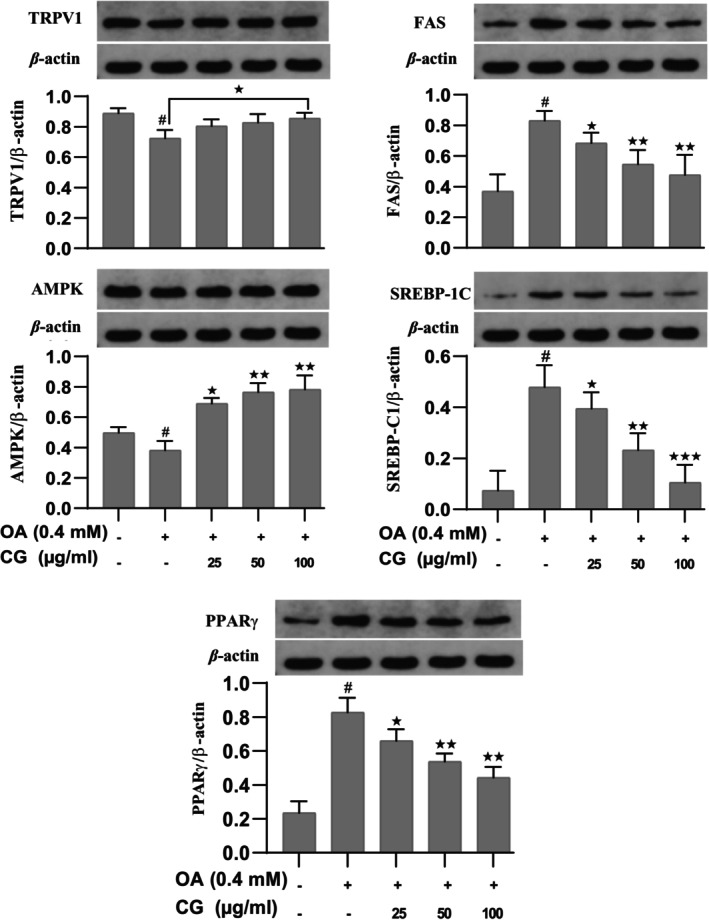
The effects of CG on protein expression related to lipid metabolism in HepG2 cells. HepG2 cells were induced, and then the mRNA expression was analyzed by real time‐PCR. All experimental data are expressed as means ± SD of at least three independent experiments. *p < 0.05 vs. eG2 cells control group, ^#^ *p* < 0.05 vs. OA group. *p < 0.05, and **p < 0.01.

Western blot analysis confirmed that CG upregulated TRPV1 and AMPK protein expression compared to the OA group (*p* < 0.05). Additionally, CG downregulated FAS and SREBP‐1C, further supporting its role in inhibiting lipogenesis (Figure 4).

### 3.8 Molecular Modeling Docking

**FIGURE 5:**
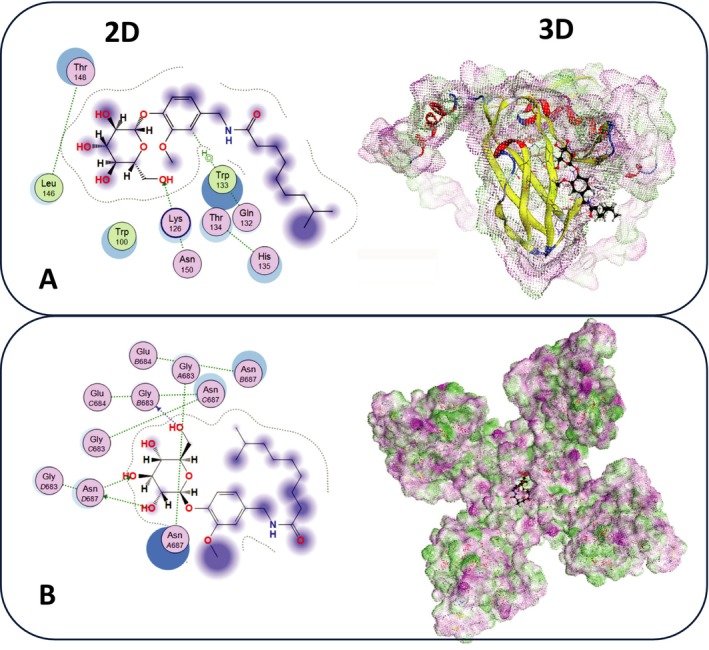
Molecular docking studies of capsaicinoid glucosides (CG) with (A) AMPK and (B) TRPV1. Each figure includes binding sites, 2D and 3D structures of the protein‐CG complexes.

Molecular docking analysis was performed to investigate the binding interactions of CG with two target proteins: AMPK and TRPV1. The results revealed that CG forms significant binding interactions with AMPK, as illustrated in the 3D structure (Figure 5B). Key residues involved in the binding site included TRP133 and LYS126. Similarly, the docking of CG with TRPV1 demonstrated interactions with residues such as VAL430, ILE433, and TYR441 (Figure 5A). The 3D structure further emphasizes the presence of both electrostatic and hydrophobic interactions.

## 4 Discussion

Obesity is a significant risk factor for metabolic disorders and a major global public health issue (GBD 2015 Obesity Collaborators 2017). Sedentary lifestyles and overeating are two primary causes of obesity. The progression of hepatic steatosis to NAFLD is accelerated by oxidative stress and lipid peroxidation. Dietary capsaicin has been linked to anti‐obesity effects by lowering body weight and fat mass, which is frequently interpreted as a result of metabolic effects that improve energy metabolism and thermogenesis (Westerterp‐Plantenga et al. 2006). In this study, we assessed the effect of capsaicinoids Glucoside on the accumulation of hepatic lipids and outlined the cellular signaling pathways. CG significantly reduces TG and TC levels in OA‐treated HepG2 cells. HepG2 cells, a well‐established in vitro model for studying lipid metabolism, exhibited a significant increase in TG and TC levels following OA treatment. The increase in lipids demonstrates that OA effectively induces lipid accumulation in HepG2 cells, making them an appropriate model for assessing the lipid‐lowering effects of CG. Treatment with CG (100 μg/mL) resulted in a significant decrease in TG and TC levels in OA‐treated HepG2 cells. CG administration alleviates ROS production in HepG2 cells by decreasing fat synthesis and boosting lipolysis via synergistic multiple signaling pathways.

Recent studies highlight the role of AMP‐activated protein kinase (AMPK) in phosphorylating nuclear factor erythroid 2‐related factor 2 (Nrf2), promoting its nuclear translocation (Elkhedir et al. 2025; Xu et al. 2020). Capsaicin has been demonstrated to reduce lipid accumulation in HepG2 cells through the activation of AMPK and PGC‐1, coupled with the inhibition of the ACC and AKT/mTOR signaling pathways (Bort et al. 2019). Furthermore, capsaicin has been found to diminish lipid content in HepG2 cells by upregulating AMPK and PPARγ activity, alongside elevating the expression levels of TRPV1 and PPARγ proteins. TRPV1, also known as vanilloid receptor 1, is a ligand‐gated ion channel belonging to the TRPV subfamily of the transient receptor potential (TRP) family. Although its role in lipid metabolism has been explored, the precise mechanisms underlying its activity and its interactions with lipid pathways remain incompletely understood (Abdalla et al. 2022). Additionally, capsaicin decreases lipid content in HepG2 cells by activating AMPK and PPARγ, while also increasing the protein levels of TRPV1 and PPARγ.

**FIGURE 6:**
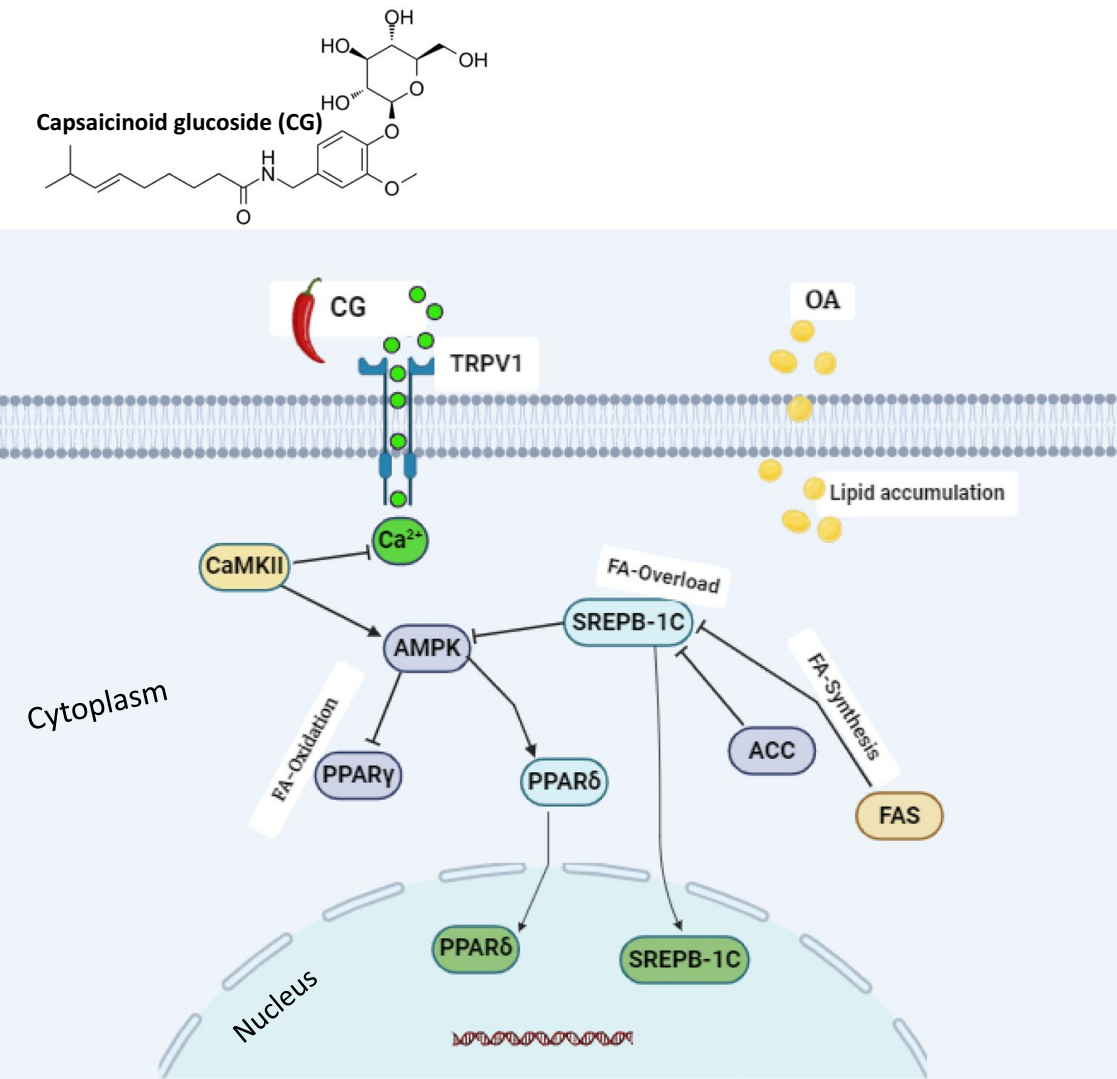
Schematic illustration highlighting the consequences of TRPV1 activation in oxidative stress induced by H_2_O_2_ metabolism. Activation of TRPV1 by CG plays a critical role in the regulation stress metabolism genes, including activation of Nrf2 and downstream genes by upregulation of AMPK.

In this study, we provide evidence that capsaicinoids suppress OA‐induced lipid accumulation by activating TRPV1, which subsequently upregulates AMPK expression in conjunction with CaMKKβ activation in response to elevated calcium levels (Figure 6). One of the most compelling mechanistic features of capsaicinoid glucoside (CG) lies in its ability to activate the TRPV1 (transient receptor potential vanilloid 1) channel, a non‐selective cation channel that responds to various vanilloids, including capsaicin. TRPV1 activation initiates intracellular Ca^2+^ influx, which serves as a second messenger in diverse signaling cascades. In hepatocytes, this calcium influx stimulates Ca^2+^/calmodulin‐dependent protein kinase kinase β (CaMKKβ), a potent upstream kinase responsible for AMPK phosphorylation and activation, particularly in LKB1‐deficient models such as HepG2 cells (Ma et al. 2012). This calcium‐triggered TRPV1/CaMKKβ/AMPK axis is emerging as a central node linking sensory receptor activation with metabolic remodeling, particularly under lipotoxic stress conditions. Upon activation, AMPK (AMP‐activated protein kinase) acts as a master metabolic switch that inhibits anabolic energy‐consuming processes while promoting catabolic energy‐generating pathways. In the context of lipid homeostasis, AMPK suppresses de novo lipogenesis by inhibiting SREBP‐1c (sterol regulatory element‐binding protein‐1c), a transcription factor that governs the expression of lipogenic enzymes including FASN (fatty acid synthase) and ACC (acetyl‐CoA carboxylase). This suppression leads to decreased synthesis of fatty acids and triglycerides. Moreover, AMPK activation leads to the disinhibition of CPT1 (carnitine palmitoyltransferase 1) by lowering malonyl‐CoA levels, thus facilitating mitochondrial β‐oxidation (Chang et al. 2013). These dual actions lipogenesis—inhibition and lipid catabolism promotion underlie—the significant reductions in triglyceride (TG) and total cholesterol (TC) levels observed in CG‐treated HepG2 cells challenged with oleic acid (OA).

In addition to its role in metabolic reprogramming, AMPK also participates in antioxidant defense through regulation of the Nrf2 (nuclear factor erythroid 2–related factor 2) pathway. Under oxidative stress, AMPK‐mediated phosphorylation facilitates Nrf2 translocation to the nucleus, where it enhances the transcription of cytoprotective genes, such as HO‐1, NQO1, and GCLC (Xu et al. 2020). Our findings that CG reduced ROS levels in OA‐treated HepG2 cells emphasize its dual antioxidant and lipid‐lowering potential, further confirming the critical involvement of the TRPV1/AMPK/Nrf2 axis in hepatocellular protection.

Another essential downstream component of this regulatory network is PPARδ (peroxisome proliferator‐activated receptor delta), a nuclear receptor whose activation has been shown to stimulate fatty acid oxidation, increase energy expenditure, and promote mitochondrial biogenesis (Lefebvre et al. 2006; Pawlak et al. 2015). In our study, CG significantly upregulated PPARδ expression while downregulating PPARγ, a receptor typically associated with adipogenesis and lipid accumulation. This expression profile suggests a favorable metabolic shift toward lipid clearance and away from storage. Activation of the PPARδ receptor has been shown to be effective in lowering TG levels in hepatocytes (Lee et al. 2012). The interplay between AMPK and PPARδ is critical in modulating transcriptional programs that support lipolysis, thermogenesis, and fatty acid catabolism, all of which contribute to hepatoprotection under lipotoxic conditions (Roberts et al. 2011).

To provide further molecular insight, in silico docking studies confirmed that CG binds specifically and stably to both AMPK and TRPV1. For AMPK, CG interacts with catalytically relevant residues TRP133 and LYS126, which are essential for allosteric activation and stability of the kinase domain (Fuertes‐Agudo et al. 2023). For TRPV1, CG demonstrates strong binding to VAL430, ILE433, and TYR441, residues critical for vanilloid ligand recognition and channel gating (Elokely et al. 2016). These structural findings reinforce the dual‐binding capability of CG, suggesting that it may concurrently initiate TRPV1 activation and AMPK phosphorylation, thereby amplifying metabolic signaling. This dual‐target mechanism adds a novel dimension to CG's bioactivity, positioning it as a multi‐modal metabolic modulator with potential therapeutic applications in obesity‐related hepatic disorders.

## 5 Conclusion

In summary, this study demonstrates that capsaicinoid glucoside (CG) effectively reduces hepatic lipid accumulation and oxidative stress in oleic acid (OA)‐treated HepG2 cells by lowering total cholesterol (TC) and triglyceride (TG) levels. These anti‐lipogenic effects are mediated through activation of the TRPV1/AMPK signaling pathway, leading to downregulation of key lipogenic genes such as SREBP‐1C and FAS. Molecular docking analysis further supports CG's potential to interact with TRPV1 and AMPK, offering mechanistic insight into its lipid‐regulating activity. Together, these findings suggest that CG holds promise as a bioactive compound for managing obesity‐related metabolic disorders, including NAFLD and dyslipidemia. However, further in vivo studies and direct molecular validation are needed to confirm these effects and advance CG toward therapeutic application.

## Author Contributions

**Abdeen Elkhedir:** investigation, data curation, conceptualization, formal analysis, methodology, resources, software, validation, visualization, writing – original draft, writing – review and editing. **Alsadig Yahya:** investigation, methodology, software, visualization, writing – review and editing. **Eihab H. Jadelrab** and **Yassin Haran:** visualization, investigation, methodology, writing – review and editing. **Amgad Albahi:** data curation, formal analysis, writing – original draft, writing – review and editing. **Mamoun A. Homaida:** data curation, formal analysis, writing – original draft, writing – review and editing. **Hammad Hamed:** visualization, formal analysis, writing – review and editing, final approval. **Mazin Mohamed Salih:** visualization, methodology, writing – original draft, writing – review and editing. **Xiaoyun Xu:** writing – review and editing.

## Supporting information


Data S1.

## Data Availability

Data will be made available upon request.
