# Supplementary material for: Capsaicinoid Glucoside Attenuates Lipid Accumulation in HepG2 Cells Through TRPV1/AMPK‐Dependent Signaling Pathway
Source: Food Sci Nutr. 2025 Jul 2;13(7):e70564. doi: 10.1002/fsn3.70564 (PMC12221993; doi:10.1002/fsn3.70564)
Supplement: Supplementary file 1 — Data S1. [file FSN3-13-e70564-s001.docx]

**CG (µg/mL)**

**Fig. S1.** Effect of capsaicinoid derivatives on HepG2 cell viability. HepG2 cells were treated with capsaicinoid glucoside (CG) at different concentrations (25, 50, 75, 100, 125 and 150) μg/mL for 24 h. Data were presented as the mean value±SD (n≥5).

**Fig. S2.** Effect of CG on ROS activities induced by OA. HepG2 cells were induced by OA and regulated by CG, and then the intracellular ROS accumulation was determined. (A) OA-induced ROS in HepG2 cells stained by fluorescent probe (DCFH-DA) and observed under fluorescence microscope. (B) Quantitative analysis of fluorescence using Image J software. (C) Fluorescence intensity of DCFH-DA on microplate reader at an excitation/emission wavelength of 485 and 528 nm. All experimental data are expressed as means ± SD of at least three independent experiments. *P< 0.05 vs OA group, # P< 0.05 vs hepG2 cells control.


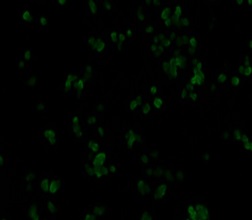

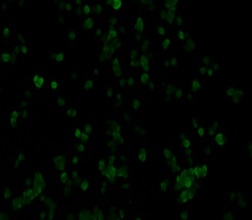

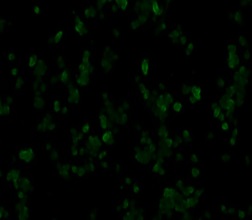

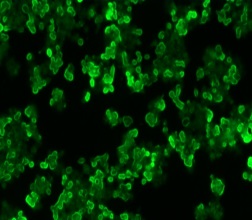

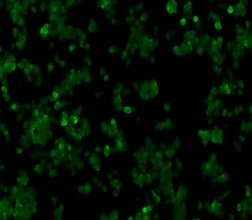


**OA (0.4 mM)**

**-**

**+**

**+**

**+**

**+**

**CG (µg/mL)**

**-**

**-**

**25**

**50**

**100**

**B**

**A**

**C**

**Fig. S3.** The effects of CG on mRNA gene expression related to lipid metabolism in HepG2 cells. HepG2 cells were induced, and then the mRNA expression of TRPV1 and AMPK was analyzed by real-time-PCR. All experimental data are expressed as means ± SD of at least three independent experiments. #P< 0.05 vs HepG2 cells control group and *P< 0.05 vs OA group.
